# ISMB 2026 Proceedings

**DOI:** 10.1093/bioinformatics/btag279

**Published:** 2026-07-07

**Authors:** Jian Ma, Karsten Borgwardt

**Affiliations:** Ray and Stephanie Lane Computational Biology Department, School of Computer Science, Carnegie Mellon University, Pittsburgh, PA 15217, United States; Department of Machine Learning and Systems Biology, Max Planck Institute of Biochemistry, 82152 Martinsried, Germany

## Abstract

This editorial describes the review and selection process of full-length, original research papers submitted to the Proceedings track of the ISMB 2026 conference.

This special issue of the *Bioinformatics* journal serves as the Proceedings of the 34th annual conference on Intelligent Systems for Molecular Biology. This meeting took place from 12 to 16 July 2026 in Washington, DC. ISMB is the leading international forum for presenting new research results, disseminating methods, and facilitating scientific discussions among researchers in the field of computational biology. ISMB is the flagship conference of the International Society for Computational Biology (ISCB) and the largest event of the computational biology community. The 2026 meeting was run as a hybrid conference, with online participants from around the world complementing the on-site participants.

A total of 408 Proceedings submissions were received and went into full review for ISMB 2026. During the submission process, the authors could indicate up to three preferred Communities of Special Interest (COSIs; Table 1; https://www.iscb.org/about-iscb/cosis) that would provide the best forum for the presentation of their paper if selected for the conference Proceedings. The aforementioned 408 submissions were thoroughly reviewed, receiving 4.30 reviews per paper on average, for a total of 1755 returned reviewer reports. For review purposes, the submitted papers were assigned to one of 11 scientific areas (Table 2) according to authors’ preferences based on their papers’ research topics, allowing for minor adjustments, e.g. to avoid conflicts of interest.

**Table 1:** COSI distribution of the accepted Proceedings papers.

COSI	Number of papers
3DSIG	8
BioInfo-Core	0
BioVis	0
BOKR	0
BOSC	0
CAMDA	0
CompMS	2
CSI	1
Education	1
EvolCompGen	4
Function	4
HitSeq	9
iRNA	1
JPI	0
Microbiome	4
MLCSB	9
NetBio	4
RegSys	4
SysMod	0
Text Mining	3
TransMed	0
VarI	0
General Comp Bio	11

**Table 2:** Thematic areas of Proceedings submissions.^a^

Area	Area Chairs	#Sub.	#Acc.	Acc.%
Bioinformatics Education and Citizen Science	Russell Schwartz, Carnegie Mellon University, USA Jason Williams, Cold Spring Harbor Laboratory, USA.	2	1	50%
Bioinformatics of Microbes and Microbiomes	Nicola Mulder, University of Cape Town, South Africa Yuzhen Ye, Indiana University, USA.	22	3	14%
Biomedical Informatics	Maria Brbic, École Polytechnique Fédérale de Lausanne, Switzerland Aasa Feragen, Technical University of Denmark, Denmark Sai Zhang, Yale University, USA.	75	11	15%
Equity and Diversity in Computational Biology Research	Anne-Christin Hauschild, Justus-Liebig-Universität Gießen, Germany Kana Shimizu, Waseda University, Japan.	4	2	50%
Evolutionary, Comparative, and Population Genomics	Flora Jay, Université Paris-Saclay, CNRS, INRIA, LISN, France Weiwei Zhai, Chinese Academy of Sciences, China.	22	5	23%
Genome Sequence Analysis	Tobias Marschall, Heinrich Heine University Düsseldorf, Germany Yun William Yu, Carnegie Mellon University, USA.	45	9	20%
Macromolecular Sequence, Structure, and Function	Minkyung Baek, Seoul National University, South Korea David Koes, University of Pittsburgh, USA Martin Steinegger, Seoul National University, South Korea.	74	12	16%
Privacy and Security for Computational Biology	Hoon Cho, Yale University, USA Gamze Gursoy, Columbia University, USA.	6	1	17%
Regulatory and Functional Genomics	Kimberly Glass, Harvard Medical School, USA Xiaowo Wang, Tsinghua University, China.	70	11	16%
Systems Biology and Networks	Anaïs Baudot, Aix Marseille Université, INSERM, CNRS, Marseille, France Vicky Yao, Rice University, USA.	50	4	8%
General Computational Biology	Jingyi Jessica Li, Fred Hutchinson Cancer Center & University of Washington, USA Rob Patro, University of Maryland, USA.	38	6	16%

a For each area, the table lists the Area Chairs, the number of submissions, the number of accepted papers, and the acceptance rate. Note that the *General Computational Biology* area was intended for submissions on emerging topics or for those papers that did not fit well in the other areas.

The review process was overseen by the Proceedings Chairs (these editorial’s authors). Each area had Area Chairs (ACs) assigned to it (Table 2), who handled the review process within their respective areas. The ACs were nominated by the ISMB 2026 Steering Committee or by COSIs. The ACs were responsible for recruiting the Program Committee for their area. Reviewers (Program Committee members, who potentially appointed subreviewers to their assigned papers) judged the papers based on the novelty of computational approaches, relevance of biological questions, importance of biological insights, clarity of presentations, expected impact, compatibility with the conference format, and, importantly, correctness, completeness, and reproducibility of the studies. After submitting their reviews, the reviewers had the opportunity to discuss the papers and refine the reviews. The ACs facilitated these discussions. Conditional/provisional acceptance decisions were made based on the reviews, the outcome of these discussions, and the outcome of another detailed discussion between the ACs and Proceedings Chairs.

Of all Proceedings submissions, 65 were provisionally accepted, conditioned on the authors properly addressing the ACs’ and reviewers’ comments during a 2-week revision period. In a few cases, the authors had to be reminded to release their source code alongside their papers or clarify some aspects of their evaluations. This year, all of the revised conditionally accepted papers were subsequently judged to have adequately addressed the ACs’ and reviewers’ concerns. These 65 papers were thus officially accepted in the end for presentation at ISMB 2026 and publication in the conference Proceedings, resulting in an overall acceptance rate of 65/408=16%. The acceptance rates for the 11 individual areas are shown in Table 2. The accepted papers were then assigned to COSIs based on the preferences of both authors and COSI organizers (Table 1). The selected papers covered a broad spectrum of topics, spanning 14 of the 23 COSIs (Table 1) and all 11 areas (Table 2).

Throughout the review process, we followed a stringent policy to avoid conflicts of interest. Submissions co-authored by an AC were reassigned to a different area. Submissions with any other type of association to an AC were handled by a different AC. Care was taken not to assign papers to reviewers with an actual or perceived conflict either. The definition of a conflict was as broad as possible, including collaborators (present and past few years), same institution (present, past few years, and planned future moves), family relations, advisees/advisors, and any personal conflicts that could cause the appearance of a conflict of interest or that could genuinely interfere with objective reviewing. Finally, as Proceedings Chairs, we refrained from submitting papers to the conference.

We are extremely grateful to the 24 ACs, 443 Program Committee members, and 546 subreviewers for their outstanding efforts in conducting thorough and timely reviews. Their contribution is at the core of the scientific quality of the conference. We also thank ISCB’s Belinda Hanson, Seth Munholland, and Diane Kovats for their support and guidance with handling many logistical aspects and the ISMB 2026 Steering Committee for their expert advice and oversight. We also thank the team at Oxford University Press for producing this special issue.

Finally, we thank all the authors for submitting their work. These Proceedings would not be possible without the scientific ingenuity of the contributors to all the papers. Despite the efforts taken to ensure fairness and high quality in the paper review and selection process, we acknowledge that the process is necessarily imperfect. Especially given the highly competitive nature of paper selection at ISMB 2026, even some very good submissions could not be accepted to the conference. Nonetheless, we hope that all authors received helpful feedback on their work. Finally, we want to thank all the keynote speakers, presenters, and all conference participants.

Thank you all for making this meeting possible and thus for empowering the ISMB community to continue to thrive.

## Conflicts of interest

K.B. is cofounder and scientific advisory board member of Computomics GmbH, Tübingen, Germany.

## Funding

None declared.

